# Fine mapping of the *Hairy glume (Hg)* gene in a chromosome variation region at the distal terminus of 1AS

**DOI:** 10.3389/fpls.2022.1006510

**Published:** 2022-09-20

**Authors:** Wei Luo, Jieguang Zhou, Jiajun Liu, Yanlin Liu, Yang Mu, Huaping Tang, Qiang Xu, Mei Deng, Qiantao Jiang, Guoyue Chen, Pengfei Qi, Jirui Wang, Yunfeng Jiang, Zhongxu Chen, Zhi Zheng, Yuming Wei, Youliang Zheng, Xiujin Lan, Jian Ma

**Affiliations:** ^1^State Key Laboratory of Crop Gene Exploration and Utilization in Southwest China, Sichuan Agricultural University, Wenjiang, Chengdu, Sichuan, China; ^2^Triticeae Research Institute, Sichuan Agricultural University, Chengdu, Sichuan, China; ^3^College of Life Science and Technology, Xinjiang University, Ürümqi, Xinjiang, China; ^4^Department of Life Science, Chengdu Tcuni Technology, Chengdu, Sichuan, China; ^5^Agriculture and Food, Commonwealth Scientific and Industrial Research Organisation, St. Lucia, QLD, Australia

**Keywords:** fine mapping, BSE-Seq, hairy glume, wheat, presence-absence variation

## Abstract

Trichomes are differentiated epidermal cells and exist on above-ground organs of nearly all land plants with important roles in resistance to a wide range of biotic and abiotic stresses. We attempted to obtain candidate gene (s) for *Hairy glume* (*Hg*), responsible for the trichome on wheat glume, by using bulked segregant exome capture sequencing (BSE-Seq), while *Hg* was only mapped in 0.52–3.26 Mb of 1AS. To further fine map this gene and identify candidate genes in this region, a near isogenic line-derived population consisting of 2,050 F_2_ lines was generated in the present study. By analyzing this population, *Hg* was fine mapped into a 0.90 cM region covering a physical distance of ~825.03 Kb encompassing 6 high- and 23 low-confidence genes in the reference genome of Chinese Spring. A presence-absence variation was identified in the fine mapping region through analyses of sequence-tagged sites markers and genome sequences of the hairy glume parent of the near isogenic lines. The results presented here will be useful for further cloning *Hg* in wheat.

## Introduction

Trichome (hair or pubescence) is a kind of unicellular or multicellular appendage with diverse functions on the plant epidermis in different organs (Werker, 2000). Previous studies showed that trichomes play important roles in resistance to a wide range of both biotic and abiotic stresses (Bickford, 2016; Galdon-Armero et al., 2018) as well as in plant defense regard to phytophagous insects (Handley et al., 2005). Additionally, hairy leaf sheath was significantly and positively correlated with increased grain yield, grain weight and grain weight per spike (Wan et al., 2015). However, glume trichome is undesirable based on some recent studies as it may be harmful to human health. It has been reported glume trichome is associated with the increased risk of developing esophageal squamous cell carcinoma following the consumption of contaminated wheat flour (Lian et al., 2019). In addition, it generates dust during harvesting and grain processes causing acute and chronic irritation to eyes, skin, and respiratory systems (Zhang et al., 2012).

QTL mapping has been routinely used to identify initial genomic regions affecting specific traits. In wheat, eight QTL conferring glume trichome have been identified. A genome-wide association study for 404 Indian bread wheat genotypes identified six putative QTL conferring hairy glume on 1A (at 292 and 511 Mb), 1B and 2B (Sheoran et al., 2019). Wu et al. (2021) reported a hairy glume gene on 2BL which was named *Hg2*. *Hg* has been long recognized as the dominant gene for glume hairiness (Kadam, 1936; Tsunewaki, 1961; Dubcovsky and Dvorák, 1995; Khlestkina et al., 2006) and it was initially mapped in a 3.3 cM region in our previous study (Luo et al., 2016). However, QTL mapping only provides limited resolution (Paterson et al., 1988) and molecular markers obtained from such studies can not be reliably used to tag targeted loci. The heterogeneity in genetic background in such mapping populations also makes accurate phenotyping of many quantitative traits difficult (Jiang et al., 2019). Therefore, fixing genetic backgrounds in regarding to a targeted locus becomes efficient for accurate phenotyping and can be achieved with near isogenic lines (NILs) or populations derived from NILs.

To further characterize *Hg*, a lot of efforts have been made including developing NILs and generating RNA sequences. In our previous study, seven pairs of NILs targeting *Hg* have been developed and difference between the two isolines was only detected on glume hairiness (Luo et al., 2020). Taken advantage of the unique feature of NILs where phenotypic difference between the two isolines of NILs mainly depends on the difference between their genomes at target locus, we also adopted multi-NIL RNA-Seq approach (Habib et al., 2017; Gao et al., 2019) to generate and analyze RNA sequences against two of the seven NIL pairs. This approach allowed us to reduce the number of candidate genes and identified 37 differentially expressed genes (DEGs) and 39 SNPs in the target region (Luo et al., 2020).

Cost of high throughput sequencing rapidly decreasing made genomic data across hundreds or even thousands of genotypes available. For example, Guo et al. (2020) released draft genome sequence of Tibetan semi-wild wheat (*Triticum aestivum* ssp. *tibetanum* Shao) and resequencing data of 245 wheat accessions. Genomic data of 641 wheat accessions were available on SnpHub (Wang et al., 2020). A recent study generated ten chromosome pseudomolecule and five scaffold assemblies of hexaploid wheat (Walkowiak et al., 2020). These data would be greatly helpful to explore large panels of high-quality genomic variation data and provide valuable resources for designing markers, identifying trait-related genes, and molecular breeding. Glume trichome in wheat has been studied for a century (Wu et al., 2021), while *Hg* has not been fine mapped. It is an opportunity to analyze it with the explosive sequencing data. Together with the available transcriptomic and genomic data, we also explored candidate region of *Hg* by bulked segregant exome capture sequencing (BSE-Seq) analysis. By developing and exploiting a NIL-derived population consisting of 2,050 F_2_ lines, we aimed to fine map *Hg* in the present study.

## Materials and methods

### Plant materials

**Figure 1:**
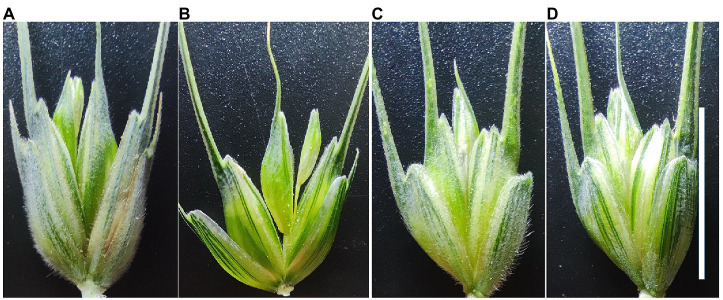
Phenotypes of the glume with or without trichome. The four glumes are **(A)** ‘3B1A’, **(B)** ‘Baiying1’, **(C)** hairy iso-line of NIL1, and **(D)** non-hairy iso-line of NIL1, respectively. Scale bar = 1 cm.

Firstly, a population of 260 F_2_ plants segregating at *Hg* generated by crossing a hairy wheat ‘3B1A’ (Figure 1A) with a non-hairy wheat accession ‘Baiying1’ (Figure 1B) was used for genetic analysis of *Hg*. These F_2_ plants were harvested and further cultivated (20 seeds of each line), and the trichome trait for each plant was investigated for confirming the phenotype of each line.

Secondly, fifty hairy and fifty non-hairy lines were randomly selected from a population of recombinant inbred lines (RILs) derived from the cross between Tibetan semi-wild wheat (*Triticum aestivum* ssp. *tibetanum* Shao) ‘Q1028’ (hairy glume) and variety ‘Zhengmai9023’ (non-hairy glume) from a previous study (Luo et al., 2016) to confirm the initial location of *Hg* and refine makers flanking the targeted region in this study. In addition, seven pairs of NILs about glume trichome were also developed from the RIL population in a previous study (Luo et al., 2020). In the present study, an F_2_ population consisting of 2,050 plants was generated by crossing the two isolines of NIL1 (Figures 1C,D) to fine map *Hg*.

### Phenotyping

The 260 F_2_ plants of ‘3B1A’ × ‘Baiying1’ materials were planted in 1.5 m rows with 0.3 m space between rows in Wenjiang experimental farm of the Triticeae Research Institute, Sichuan Agricultural University, China. The 2,050 F_2_ plants derived from the crossing of the two isolines of NIL1 were cultivated in the 5 × 10 plug tray with 50 cm in length × 28 cm in width × 10 cm in height. The tray was placed in the greenhouse (constant temperature at 22°C, light for 16 h and darkness for 8 h) of Triticeae Research Institute, Sichuan Agricultural University. The phenotypes of wheat glumes were investigated and recorded as hairy or non-hairy.

### BSE-Seq analysis

The approximately equal leaf of 32 homozygous hairy and 32 non-hairy F_3_ lines of ‘3B1A’ × ‘Baiying1’ were sampled and pooled separately. DNA of the two pools and parents were extracted using the cetyltrimethylammonium bromide method. Exome capture sequencing (Illumina HiSeq Nova platform) and analysis for the four libraries (‘3B1A’, ‘Baiying1’, hairy pool, and non-hairy pool) were performed by Tiancheng Weilai Technology^1^ (Chengdu, China) using a wheat exome capture panel. It contained 268.9 Mb capture space and covered 107,400 high confidence (HC) genes and 132,688 HC transcripts of International Wheat Genome Sequencing Consortium (IWGSC) Annotation v1.1 (Dong et al., 2020).

Both SNP-index and G’ methods were used to screen the significant region for bulk segregant analysis, and R package QTLseqr (Mansfeld and Grumet, 2018) was used to calculate SNP-index and G’ based on sliding window. Allelic frequency with the significance of linearity was performed using Euclidean distance (ED; Hill et al., 2013). ED value of each locus was calculated, the 4^th^ power of the original ED was set as correlation value to eliminate background noise, and Loess was used to fit the ED value (Ji et al., 2021). varBScore was also used for BSE-Seq analysis (Dong et al., 2020).

### Data collection and molecular marker development

The resequencing data of 641 wheat accessions were collected and analyzed on the database of SnpHub (Wang et al., 2020). In our previous study, 83 of the 641 accessions including 24 hairy and 59 non-hairy genotypes were observed (Supplementary Table S1; Luo et al., 2019). Resequencing data of these 83 genotypes were downloaded and used to detect the InDel and SNP variations in the candidate region.

The flanking genomic sequences of InDel (more than 14 base pairs) were used as templates to develop InDel markers. HC genes from IWGSC Annotation v1.1 identified from the initial mapping region were used to detect the sequence variants between the two isolines of the NIL1. The variations between the NIL1 were used for developing PCR-based markers, including KASP (Kompetitive Allele-Specific PCR) and CAPS (cleaved amplified polymorphic sequence) markers. Some HC and low confidence (LC) genes in the fine mapping region were used as templates to develop sequence-tagged sites (STS) markers. Primers for CAPS and KASP markers were designed on websites^2^^,^^3^ and those for the others were designed using WheatOmics 1.0 (Ma et al., 2021).

Primer synthesis and gene sequencing were completed by Tsingke Biotechnology Co., Ltd. (Chengdu, China^4^). The amplification for KASP markers were carried out with KASP-TF V4.0 2× Master Mix (LGC Genomics, Hoddeson, United Kingdom) using CFX96 Touch Real-Time PCR Detection System (Bio-Rad, Hercules, United States) according to the manufacturer’s instructions. Other markers were amplified by 2× Taq Master Mix (P213; Vazyme Biotech Co., Ltd., Nanjing, China).

### The detection of chromosome variation in the targeted region

In order to detect the chromosome variation in the fine mapping region, the STS markers were amplified in NIL1. The amplification products were analyzed by 2% agarose gel electrophoresis. Furthermore, the chromosome variation was preliminarily analyzed using the genomic sequence of the hairy glume parent of NIL1.

The 30-fold wheat whole-genome data of ‘Q1028’ was provided by Triticeae Research Institute, Sichuan Agricultural University (unpublished). The leaves of ‘Q1028’were used to extract genomic DNA using Plant Genomic DNA Kit of Tiangen Biotech Co., Ltd. (Beijing, China). Genomic DNA of ‘Q1028’ was sent to BerryGenomics (Beijing, China) for resequencing using 10× Genomics. After quality control, sequencing reads were mapped to IWGSC Reference Sequences (RefSeq) v1.0 (IWGSC, 2018) using Resequencing Analysis function of Toolbox on CLC Genomics Workbench 12.0.^5^ The parameters for mapping resequencing reads of ‘Q1028’ to reference genomes were shown in Supplementary Table S2. Then the Whole Genome Coverage Analysis was performed in the Resequencing Analysis function of Toolbox on CLC Genomics Workbench 12.0 with the following parameters: P -Value threshold = 0.0001, Minimum length = 50, Create regions = Yes, Create report = Yes.

## Results

### Genetic analysis of trichome gene

Chi-square test demonstrated that the ratio of hairy:non-hairy glume fit the 3:1 segregation ratio in the F_2_ populations of ‘3B1A’ × ‘Baiying1’ (Table 1). The 2,050 NIL-derived F_2_ plants derived from the cross between the NIL1 also displayed a 3:1 segregation ratio for the hairy and the non-hairy glume (Table 1). These results indicated that hairy glume trait was controlled by a dominant gene in ‘3B1A’ and hairy isoline of NIL1.

**Table 1:** Chi-square test for hairy:non-hairy glume.

Population	Total *F*_2_ plants	Hairy plants	Non-hairy plants	*χ^2^*
3B1A × Baiying1	260	195	65	0.01
Hairy × non-hairy isoline	2,050	1,510	540	1.90

$\chi_{0.05}^{2}$ = 3.84

### Chromosome localization of trichome gene in BSE-Seq analysis

**Figure 2:**
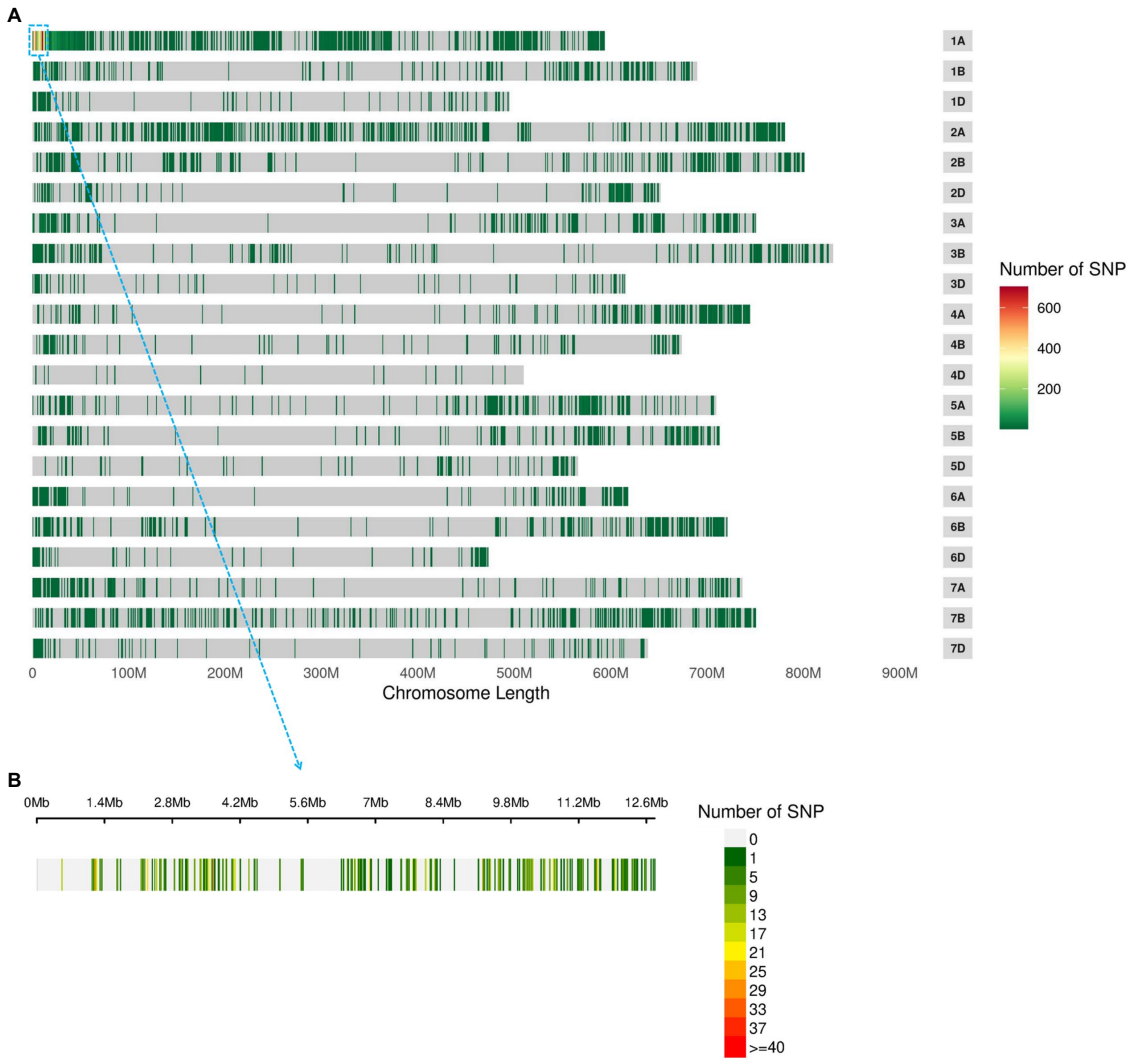
The nonsymmetrical variations of BSE-Seq analysis. The variable sites in the genome **(A)** and 1–10 Mb of chromosome 1A **(B)**.

BSE-Seq generated 29, 26, 62 and 57 Gb raw data from ‘3B1A’, ‘Baiyin1’, the hairy and non-hairy pools, respectively, and their data size reached about 109, 101, 234 and 214 times to the exome capture panel (Supplementary Table S3). The Q30 of the library was over 91% (Supplementary Table S3). Furthermore, the percentage of properly paired reads was more than 98% in each library (Supplementary Table S3). A total of 13,895 nonsymmetrical variations were obtained from the two bulked pools, and half of these variations were concentrated at chromosome 1A (Figure 2A; Supplementary Table S4). The variations at the distal terminus of 1AS was the densest, and several regions with no SNPs were detected on this interval, for example, 1.34–2.16 (only 21 sites were detected around 1.7 Mb in this gap) Mb (Figure 2B; Supplementary Table S4).

**Figure 3:**
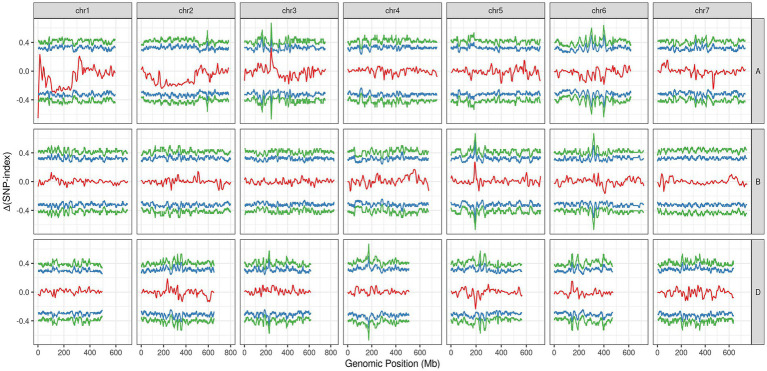
The tricube-smoothed ∆(SNP-index; red line) of BSE-Seq analysis. The blue and green lines indicated for confidence intervals of 95 and 99%, respectively.

In the varBScore analysis, the peak scoring points were distributed in three regions on chromosome 1A, namely, 1.20–1.21, 6.56–6.57 and 10.72–12.59 Mb (Supplementary Figure S1). The results of tricube-smoothed ∆(SNP-index) showed that only one region existed on 1AS (0.52–3.26 Mb) with a confidence interval of larger than 99%, indicating that a single locus for glume trichome was defined in the genome (Figure 3). A similar result was also detected in G’ analysis, and the peak values of tricube-smoothed ∆(SNP-index) and G’ were both at 521,162 bp on 1AS (Supplementary Figure S2). In the result of ED analysis, the top 1% fitted ED values were distributed in 0.52–14.08 Mb (Supplementary Figure S3), and the peak value was also at 521,162 bp. These results suggested that the candidate gene of *Hg* may be located at 0.52–3.26 Mb of 1AS, and a gap, 1.34–2.16 Mb, existed in the region.

### Identification of the genomic region containing *Hg*

**Figure 4:**
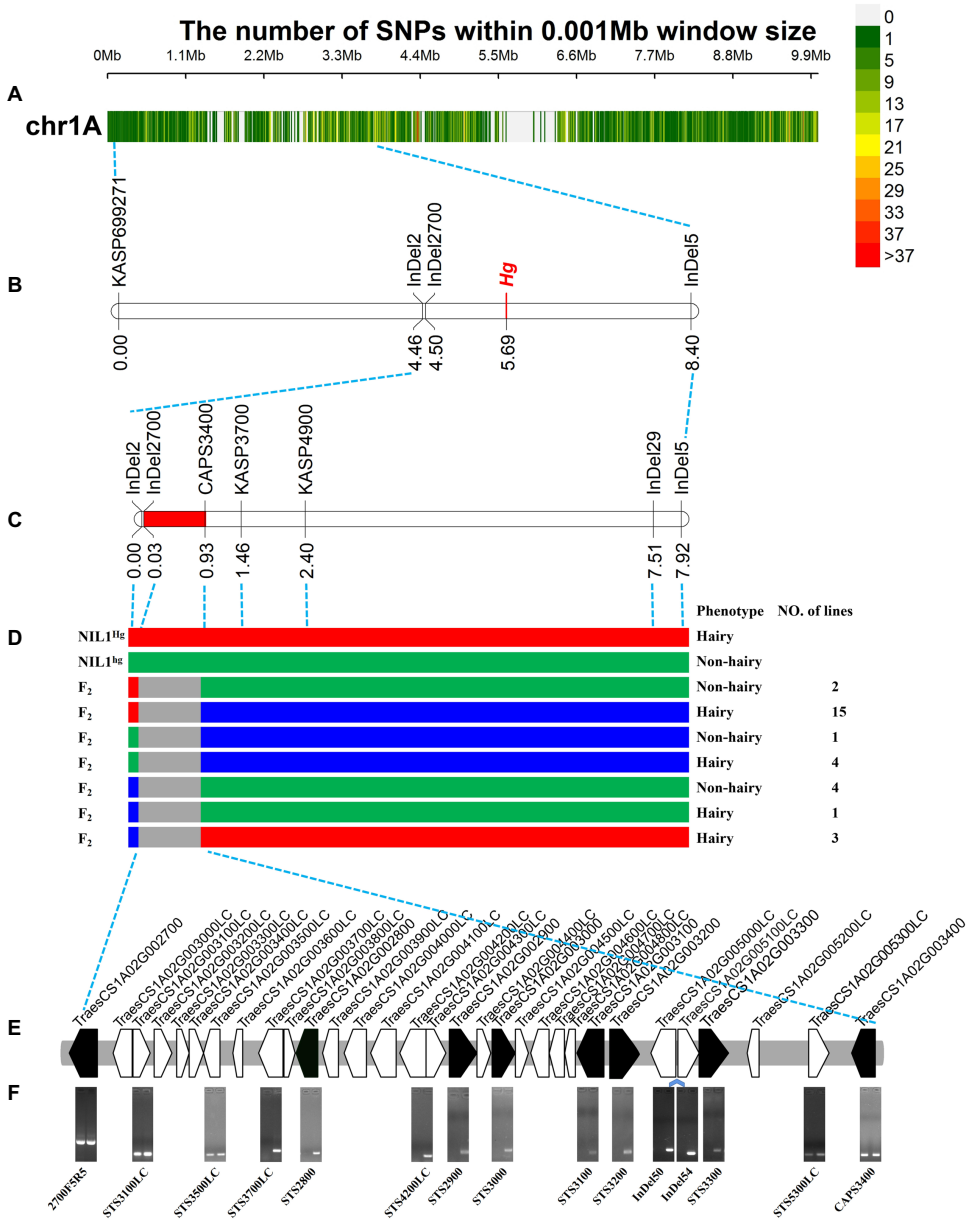
Fine mapping of *Hg*. **(A)** The variations in 0–10 Mb among the 83 released resequencing wheat accessions. **(B)** The primary mapping of *Hg* with the markers based on the resequencing data. **(C)** The fine mapping region of *Hg*. **(D)** The recombinants from the fine mapping region, the three colors indicated the genotypes of hairy (red), heterozygous (blue), and non-hairy lines (green), respectively. **(F)** The genes in fine mapping region from IWGSC Annotation v1.1. **(D)** The amplification of the primers for the genes in the mapping region. The bands of *TraesCS1A02G002700* were the amplification products using sequencing primers of 2700F5 and 2700R5; STS4200LC was designed based on the overlapping sequence of *TraesCS1A02G004200LC* and *TraesCS1A02G004300LC*; InDel50 and InDel54 were two InDel markers based on the re-sequencing data in the region between *TraesCS1A02G005000LC* and *TraesCS1A02G005100LC*; the bands of *TraesCS1A02G003400* were the amplification products of CAPS3400F and CAPS3400R (Enzyme digestion has not been carried out). The left and right lanes were the hairy and non-hairy isolines, respectively.

A total of 27,607 polymorphic sites (Figure 4A) among the 83 resequencing accessions were obtained including 22,046 SNPs and 5,561 InDels. Of them, 163 InDel markers (Supplementary Table S5) and 19 KASP markers (Supplementary Table S6) were developed and used to screen against the NIL1, and eight markers were detected polymorphism between the two isolines.

The newly developed markers were then used to screen the 100 RILs to confirm the initial location of *Hg*. *Hg* was located in the region between InDel2 and InDel5 (Figure 4B). After that, *TraesCS1A02G002700* (one of the closest gene to InDel2) was isolated based on the chromosome 1A specific primers (Supplementary Table S7) and sequenced in NIL1, and a 122 bp sequence was detected to be inserted in the hairy isoline. An InDel marker, InDel2700, was designed and mapped closer to *Hg* in the RIL population (Figure 4B).

### Fine mapping of *Hg*

Based on the positions and sequences of the two flanking markers, twelve genes (Supplementary Table S7) were sequenced from the two isolines of NIL1 and three polymorphic markers (CAPS3400, KASP3700, and KASP4900; Supplementary Table S8) were generated and used to genotype the population and construct a high-density genetic map covering the targeted region. Finally, *Hg* was placed into a 0.90 cM region between InDel2700 and CAPS3400 covering a physical region of ~825.03 Kb (1,337,248-2,162,275 bp; Figure 4C). By assessing the whole population of 2,050 lines with the two flanking markers (InDel2700 and CAPS3400), 30 recombinants were identified (Figure 4D). Phenotyping of these recombinants found that 23 were hairy and the other 7 were non-hairy (Figure 4D). Six HC and 23 LC genes (Figure 4E; Supplementary Table S8) were annotated in this fine mapping region according to IWGSC Annotation v1.1.

### A chromosome variation in the fine mapping region

In the fine mapping region, some genes can be easily amplified in non-hairy isoline of NIL1 while no bands or target sequences were obtained in the hairy isoline. For instance, the target sequence for the homolog *TraesCS1A02G003000*, was obtained in the non-hairy isolines of NIL1, while the amplified sequence from hairy isolines was more similar to the homoeolog on 1B (Supplementary Figure S4).

STS markers were then designed for the conserved region of 6 HC and 5 LC genes (Figure 4E; Supplementary Table S9). As expected, the 11 STS and 2 InDel markers were detected in non-hairy isoline of NIL1 (Figure 4F). However, the markers between *TraesCS1A02G003500LC* and *TraesCS1A02G005300LC* were not detected in hairy isoline of NIL1 (Figure 4F). These results indicated that a presence-absence variation (PAV) might exist in the fine mapping region.

**Figure 5:**
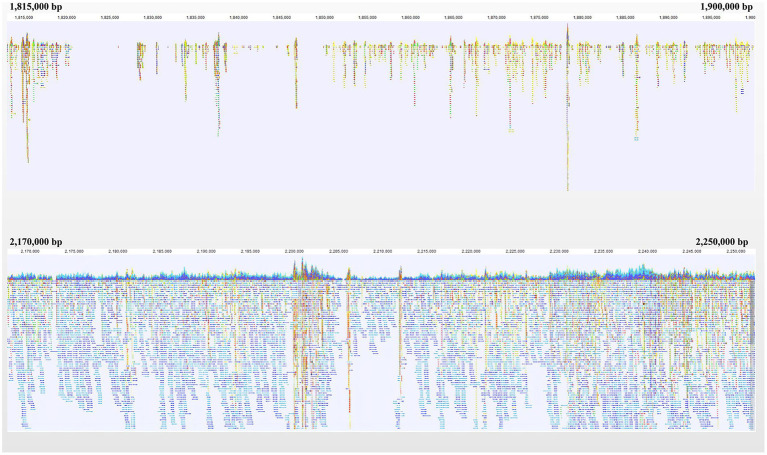
Navigation overview of two reads mapping regions on Chromosome 1A. Each color block represented a read. Each color block represents a read. The closer it is to blue or red, the more similar or different the read is to the reference, respectively.

**Figure 6:**
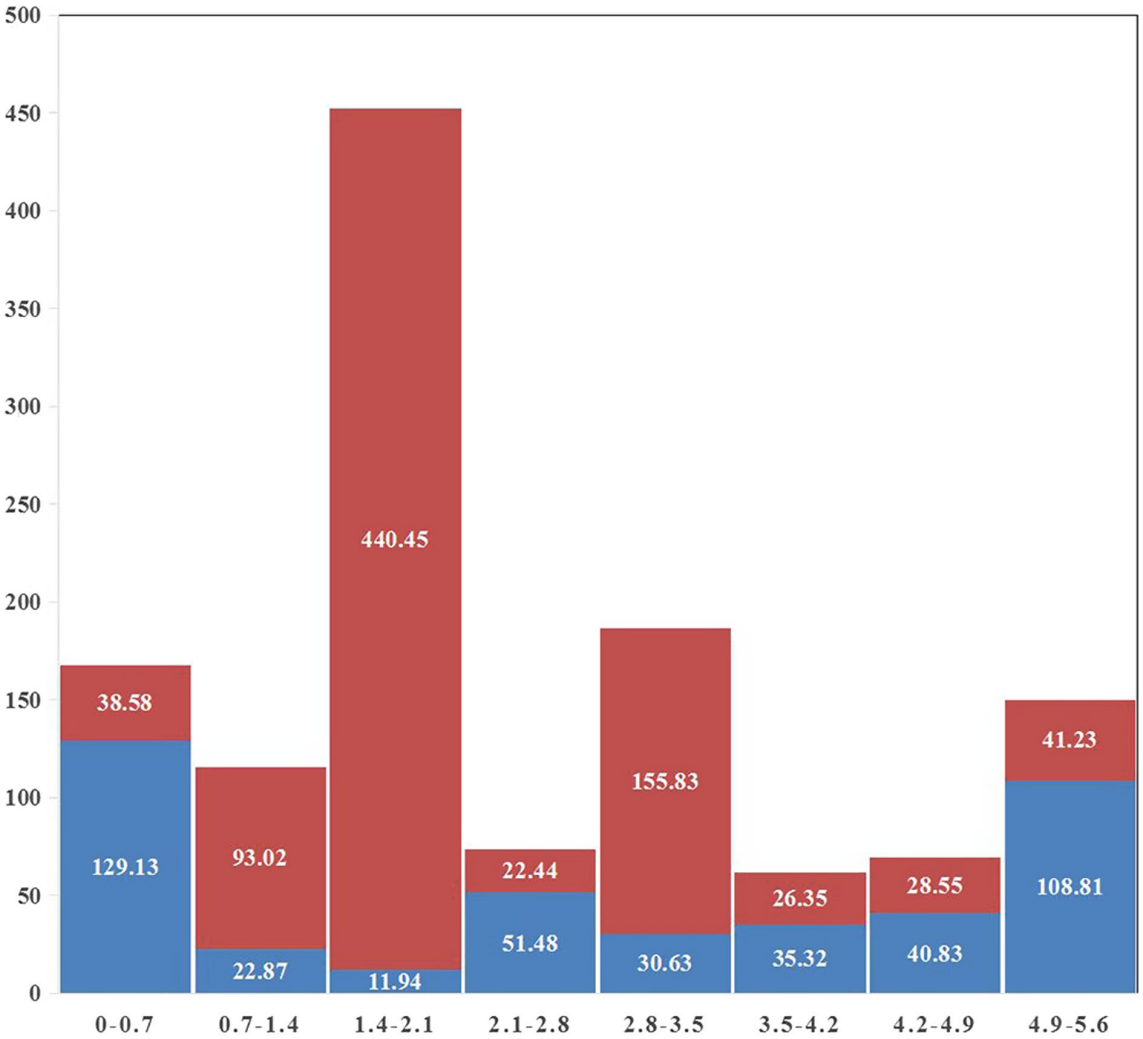
Statistics of abnormal reads coverage around fine mapping interval. Red and blue indicated low and high coverage, respectively. The abscissa was the physical map of 1A (MB) and the ordinate was the total length of abnormal reads (KB).

A gap was detected in the region from ~1.40 Mb to ~2.10 Mb when mapping the resequencing reads of Q1028 to IWGSC RefSeq v1.0. There was a low coverage region with a few reads and most of these reads showed huge difference relative to the reference sequence. For example, a gap was shown in 1.82–1.90 Mb, and a normal region was shown in 2.17–2.25 Mb (Figure 5). It was found that the number of abnormal reads in fine mapping region were larger than in the flanking regions (Figure 6). The abnormal high and low coverage reads were quite less and larger than the flanking regions (Figure 6), respectively. Taken together, the PAV was further identified through resequencing of the donor of *Hg*.

## Discussion

*Hg* as the dominant gene for glume trichome has been studied for many years. However, candidate genes underlying this locus remain unknown. In the present study, we developed and assessed a large NIL-derived population targeting this locus and finally placed this gene into a 0.90 cM genetic region corresponding to a ~825.03 Kb physical region (1,337,248–2,162,275 bp) on chromosome 1AS. A PAV was identified in the candidate region according to amplification results of STS markers and sequencing data analysis of Q1028. This PAV at the mapping region may hamper the following work of gene cloning of *Hg*.

It has been reported that prediction of candidate genes *via* fine mapping was impeded by chromosome variation, so alternative approaches have been arisen to solve the problem. Nsabiyera et al. (2019) suggested resequencing of the parents of mapping population and *de novo* assemble of the target region was the ideal way to obtain the functional genes. Lang et al. (2021) used the same method and discovered the gene for pre-harvest sprouting resistance gene from synthetic hexaploid in a PAV region on 3D. Hewitt et al. (2021) isolated the functional gene conferring powdery mildew resistance by single chromosome enrichment sequencing for 7A. These reports provided us clues to explore the candidate genes from a chromosome variation region.

Chromosome variants might be involved in crop domestication and adaptation (Huang et al., 2021). Besides the distal terminus of 1AS, chromosome variation has been reported at the distal terminus of other chromosomes. For example, a heat stress tolerance gene was mapped in the PAV region at the distal terminus of 4AL (Zhai et al., 2021). Bunt resistance gene *Bt9* was located on the distal terminus of 6DL, and PAV markers for *Bt9* selection had been identified (Morris and Beecher, 2012). Furthermore, there was a rearrangement at the distal region of chromosome 7AL (Hewitt et al., 2021). These variants indicated that chromosome extremities were the most variational regions in wheat (De Oliveira et al., 2020), which was proved by wheat-rice comparative genomics, and gene evolution appeared preferentially at chromosome extremities due to the high frequency of recombination duplication and divergence (See et al., 2006).

We found that the percentage of hairy glume in released wheat cultivars is much less than that of wild or semi-wild wheat (Luo et al., 2019). Furthermore, 35.7% of the genes in bread wheat were dispensable (Montenegro et al., 2017). It was reported that trichome is not necessary for the growth and development of the plant itself although it provides resistance to biotic and abiotic stress (Zhang et al., 2020). Trichome on wheat glume may be a dispensable structure controlled by dispensable genes, while it may provide clues for studying the evolution and domestication of wheat.

## Conclusion

In summary, *Hg* was fine mapped in a ~ 825.03 Kb physical region on chromosome 1A, and a PAV was identified in this region. Assembling of resequencing data may be an effective way to explore *Hg*.

## Data availability statement

The data presented in the study are deposited in the National Genomics Data Center (NGDC) repository, accession number PRJCA010932.

## Author contributions

WL finished the study and wrote this manuscript. JZ participated in glasshouse work, phenotype investigation, and data analysis. JL, YL, and YM helped phenotype investigation. HT helped data analysis and maker development. QX and MD collected and analyzed data. QJ, GC, and PQ helped with data analysis and revision. JW and YJ analyzed resequencing data. ZC performed BSE-Seq analysis. ZZ and YW revised the manuscript. YZ and XL discussed results. JM designed the experiments, guided the entire study, and extensively revised this manuscript. All authors participated in the research and approved the final manuscript.

## Funding

This research was funded by the National Natural Science Foundation of China (31970243 and 31971937), Sichuan Science and Technology Program (2022YFH0053, 2022NSFSC1729, and 2021YFH0083), and the Basic Research Project of Science and Technology Plan of Guizhou Province (ZK[2021] General 131). We thank the anonymous referees for critical reading and revising this manuscript.

## Conflict of interest

The authors declare that the research was conducted in the absence of any commercial or financial relationships that could be construed as a potential conflict of interest.

## Publisher’s note

All claims expressed in this article are solely those of the authors and do not necessarily represent those of their affiliated organizations, or those of the publisher, the editors and the reviewers. Any product that may be evaluated in this article, or claim that may be made by its manufacturer, is not guaranteed or endorsed by the publisher.

## Footnotes

^1^ http://www.tcuni.cn

^2^ https://galaxy.triticeaetoolbox.org/

^3^ http://www.polymarker.info/

^4^ https://tsingke.com.cn/

^5^ https://digitalinsights.qiagen.com/products-overview/discovery-insights-portfolio/analysis-and-visualization/qiagen-clc-genomics-workbench/

## Supplementary material

SUPPLEMENTARY FIGURE S1 Result of varBScore analysis for BSE-Seq. The above and below figures were drawn based on the varBScore of the variable sites of the whole genome and chromosome 1A, respectively.

SUPPLEMENTARY FIGURE S2 Tricube-smoothed G’ value for BSE-Seq.

SUPPLEMENTARY FIGURE S3 Result of ED analysis for BSE-Seq. The above and below figures were drawn based on the fitted curve and original value, respectively.

SUPPLEMENTARY FIGURE S4 Sequence comparison of amplified *TraesCS1A02G003000* in NIL1.

## References

[ref1] Bickford C. P. (2016). Ecophysiology of leaf trichomes. Funct. Plant Biol. 43, 807–814. doi: 10.1071/FP16095, PMID: 32480505

[ref3] De Oliveira R. Rimbert H. Balfourier F. Kitt J. Dynomant E. Vrana J.. (2020). Structural variations affecting genes and transposable elements of chromosome 3B in wheats. Front. Genet. 11, 891. doi: 10.3389/fgene.2020.00891, PMID: 33014014 PMC7461782

[ref4] Dong C. Zhang L. Chen Z. Xia C. Gu Y. Wang J.. (2020). Combining a new exome capture panel with an effective varBScore algorithm accelerates BSA-based gene cloning in wheat. Front. Plant Sci. 11, 1249. doi: 10.3389/fpls.2020.01249, PMID: 32903549 PMC7438552

[ref5] Dubcovsky J. Dvorák J. (1995). Ribosomal RNA multigene loci: nomads of the Triticeae genomes. Genetics 140, 1367–1377. doi: 10.1093/genetics/140.4.1367, PMID: 7498776 PMC1206700

[ref6] Galdon-Armero J. Fullana-Pericas M. Mulet P. A. Conesa M. A. Martin C. Galmes J. (2018). The ratio of trichomes to stomata is associated with water use efficiency in *Solanum lycopersicum* (tomato). Plant J. 96, 607–619. doi: 10.1111/tpj.14055, PMID: 30066411 PMC6321981

[ref7] Gao S. Zheng Z. Powell J. Habib A. Stiller J. Zhou M.. (2019). Validation and delineation of a locus conferring *Fusarium crown* rot resistance on 1HL in barley by analysing transcriptomes from multiple pairs of near isogenic lines. BMC Genomics 20, 650. doi: 10.1186/s12864-019-6011-8, PMID: 31412765 PMC6694680

[ref8] Guo W. Xin M. Wang Z. Yao Y. Hu Z. Song W.. (2020). Origin and adaptation to high altitude of Tibetan semi-wild wheat. Nat. Commun. 11, 5085. doi: 10.1038/s41467-020-18738-5, PMID: 33033250 PMC7545183

[ref9] Habib A. Powell J. J. Stiller J. Liu M. Shabala S. Zhou M.. (2017). A multiple near isogenic line (multi-NIL) RNA-seq approach to identify candidate genes underpinning QTL. Theor. Appl. Genet. 131, 613–624. doi: 10.1007/s00122-017-3023-0 29170790

[ref10] Handley R. Ekbom B. Ågren J. (2005). Variation in trichome density and resistance against a specialist insect herbivore in natural populations of *Arabidopsis thaliana*. Ecol. Entomol. 30, 284–292. doi: 10.1111/j.0307-6946.2005.00699.x

[ref11] Hewitt T. Müller M. C. Molnár I. Mascher M. Holušová K. Šimková H.. (2021). A highly differentiated region of wheat chromosome 7AL encodes a *Pm1a* immune receptor that recognizes its corresponding *AvrPm1a* effector from *Blumeria graminis*. New Phytol. 229, 2812–2826. doi: 10.1111/nph.17075, PMID: 33176001 PMC8022591

[ref12] Hill J. T. Demarest B. L. Bisgrove B. W. Gorsi B. Su Y. C. Yost H. J. (2013). MMAPPR: mutation mapping analysis pipeline for pooled RNA-seq. Genome Res. 23, 687–697. doi: 10.1101/gr.146936.112, PMID: 23299975 PMC3613585

[ref13] Huang Y. Huang W. Meng Z. Braz G. T. Li Y. Wang K.. (2021). Megabase-scale presence-absence variation with Tripsacum origin was under selection during maize domestication and adaptation. Genome Biol. 22, 237. doi: 10.1186/s13059-021-02448-2, PMID: 34416918 PMC8377971

[ref14] IWGSC (2018). Shifting the limits in wheat research and breeding using a fully annotated reference genome. Science 361:eaar7191. doi: 10.1126/science.aar7191 30115783

[ref15] Ji G. Xu Z. Fan X. Zhou Q. Yu Q. Liu X.. (2021). Identification of a major and stable QTL on chromosome 5A confers spike length in wheat (*Triticum aestivum* L.). Mol. Breed. 41:56. doi: 10.1007/s11032-021-01249-6 PMC10236030 37309397

[ref16] Jiang Y. Habib A. Zheng Z. Zhou M. Wei Y. Zheng Y.-L.. (2019). Development of tightly linked markers and identification of candidate genes for Fusarium crown rot resistance in barley by exploiting a near-isogenic line-derived population. Theor. Appl. Genet. 132, 217–225. doi: 10.1007/s00122-018-3209-0, PMID: 30327844

[ref17] Kadam B. (1936). Genetics of the Bansi wheat of the Bombay-Deccan and a synthetic Khapli-part I. Proc. Indian As. B. 4, 357–369.

[ref18] Khlestkina E. K. Pshenichnikova T. A. Roder M. S. Salina E. A. Arbuzova V. S. Borner A. (2006). Comparative mapping of genes for glume colouration and pubescence in hexaploid wheat (*Triticum aestivum* L.). Theor. Appl. Genet. 113, 801–807. doi: 10.1007/s00122-006-0331-1, PMID: 16874490

[ref19] Lang J. Fu Y. Zhou Y. Cheng M. Deng M. Li M.. (2021). *Myb10-D* confers *PHS-3D* resistance to pre-harvest sprouting by regulating *NCED* in ABA biosynthesis pathway of wheat. New Phytol. 230, 1940–1952. doi: 10.1111/nph.17312, PMID: 33651378 PMC8251712

[ref20] Lian C. Zuo X. Tian L. (2019). A possible role of biogenic silica in esophageal cancer in North China? Environ. Sci. Pollut. R. 26, 8340–8343. doi: 10.1007/s11356-019-04332-w, PMID: 30689109

[ref22] Luo W. Liu J. Ding P. Li C. Liu H. Mu Y.. (2020). Transcriptome analysis of near-isogenic lines for glume hairiness of wheat. Gene 739:144517. doi: 10.1016/j.gene.2020.144517, PMID: 32113949

[ref23] Luo W. Liu J. Ding P. Zou Y. Li T. Xie Q.. (2019). Domestication of glume hairiness in wheat. J. Sichuan Agr. Univ. 37, 743–754. doi: 10.16036/j.issn.1000-2650.2019.06.001

[ref24] Luo W. Ma J. Zhou X.-h. Jiang Y.-f. Sun M. Yang Y.-j.. (2016). Genetic analysis of glume hairiness (*Hg*) gene in bread wheat (*Triticum aestivum* L.). Genet. Resour. Crop Evol. 63, 763–769. doi: 10.1007/s10722-016-0393-0

[ref26] Ma S. Wang M. Wu J. Guo W. Chen Y. Li G.. (2021). WheatOmics: A platform combining multiple omics data to accelerate functional genomics studies in wheat. Mol. Plant 14, 1965–1968. doi: 10.1016/j.molp.2021.10.006, PMID: 34715393

[ref27] Mansfeld B. N. Grumet R. (2018). QTLseqr: an R package for bulk segregant analysis with next-generation sequencing. Plant Genome 11:180006. doi: 10.3835/plantgenome2018.01.0006 PMC12810111 30025013

[ref28] Montenegro J. D. Golicz A. A. Bayer P. E. Hurgobin B. Lee H. Chan C. K.. (2017). The pangenome of hexaploid bread wheat. Plant J. 90, 1007–1013. doi: 10.1111/tpj.13515, PMID: 28231383

[ref29] Morris C. F. Beecher B. S. (2012). The distal portion of the short arm of wheat (*Triticum aestivum* L.) chromosome 5D controls endosperm vitreosity and grain hardness. Theor. Appl. Genet. 125, 247–254. doi: 10.1007/s00122-012-1830-x, PMID: 22366813

[ref31] Nsabiyera V. Baranwal D. Qureshi N. Kay P. Forrest K. Valarik M.. (2019). Fine mapping of *Lr49* using 90K SNP chip array and flow-sorted shromosome sequencing in wheat. Front. Plant Sci. 10, 1787. doi: 10.3389/fpls.2019.01787 32117347 PMC7010802

[ref32] Paterson A. H. Lander E. S. Hewitt J. D. Peterson S. Lincoln S. E. Tanksley S. D. (1988). Resolution of quantitative traits into Mendelian factors by using a complete linkage map of restriction fragment length polymorphisms. Nature 335, 721–726. doi: 10.1038/335721a0, PMID: 2902517

[ref33] See D. R. Brooks S. Nelson J. C. Brown-Guedira G. Friebe B. Gill B. S. (2006). Gene evolution at the ends of wheat chromosomes. Proc. Natl. Acad. Sci. U. S. A. 103, 4162–4167. doi: 10.1073/pnas.0508942102, PMID: 16537502 PMC1449664

[ref34] Sheoran S. Jaiswal S. Kumar D. Raghav N. Sharma R. Pawar S.. (2019). Uncovering genomic regions associated with 36 agro-morphological traits in Indian spring wheat using GWAS. Front. Plant Sci. 10, 527. doi: 10.3389/fpls.2019.00527, PMID: 31134105 PMC6511880

[ref35] Tsunewaki K. (1961). Monosomic and conventional gene analyses in common wheat IV. Glume hairiness and ear density. JPN. J. Genet. 36, 55–62.

[ref36] Walkowiak S. Gao L. Monat C. Haberer G. Kassa M. T. Brinton J.. (2020). Multiple wheat genomes reveal global variation in modern breeding. Nature 588, 277–283. doi: 10.1038/s41586-020-2961-x, PMID: 33239791 PMC7759465

[ref37] Wan H. Yang Y. Li J. Zhang Z. Yang W. (2015). Mapping a major QTL for hairy leaf sheath introgressed from *Aegilops tauschii* and its association with enhanced grain yield in bread wheat. Euphytica 205, 275–285. doi: 10.1007/s10681-015-1457-5

[ref38] Wang W. Wang Z. Li X. Ni Z. Hu Z. Xin M.. (2020). SnpHub: an easy-to-set-up web server framework for exploring large-scale genomic variation data in the post-genomic era with applications in wheat. GigaScience 9:giaa060. doi: 10.1093/gigascience/giaa060, PMID: 32501478 PMC7274028

[ref39] Werker E. (2000). Trichome diversity and development. Adv. Bot. Res. 31, 1–35. doi: 10.1016/S0065-2296(00)31005-9

[ref40] Wu P. Yang L. Guo G. Hu J. Qiu D. Li Y.. (2021). Molecular mapping and identification of a candidate gene for new locus *Hg2* conferring hairy glume in wheat. Plant Sci. 307:110879. doi: 10.1016/j.plantsci.2021.110879, PMID: 33902847

[ref41] Zhai H. Jiang C. Zhao Y. Yang S. Li Y. Yan K.. (2021). Wheat heat tolerance is impaired by heightened deletions in the distal end of 4AL chromosomal arm. Plant Biotechnol. J. 19, 1038–1051. doi: 10.1111/pbi.13529, PMID: 33372381 PMC8131055

[ref42] Zhang Y. Song H. Wang X. Zhou X. Zhang K. Chen X.. (2020). The roles of different types of trichomes in tomato resistance to cold, drought, whiteflies, and botrytis. Agronomy 10, 411. doi: 10.3390/agronomy10030411

[ref43] Zhang H. Wu K. Wang Y. Peng Y. Hu F. Wen L.. (2012). A *WUSCHEL*-like homeobox gene, *OsWOX3B* responses to *NUDA/GL-1* locus in rice. Rice 5, 30. doi: 10.1186/1939-8433-5-30, PMID: 27234248 PMC5520835

