# Supplementary figures and images for: Fine mapping of the Hairy glume (Hg) gene in a chromosome variation region at the distal terminus of 1AS
Source: Front Plant Sci. 2022 Sep 20;13:1006510. doi: 10.3389/fpls.2022.1006510 (PMC9530909; doi:10.3389/fpls.2022.1006510)

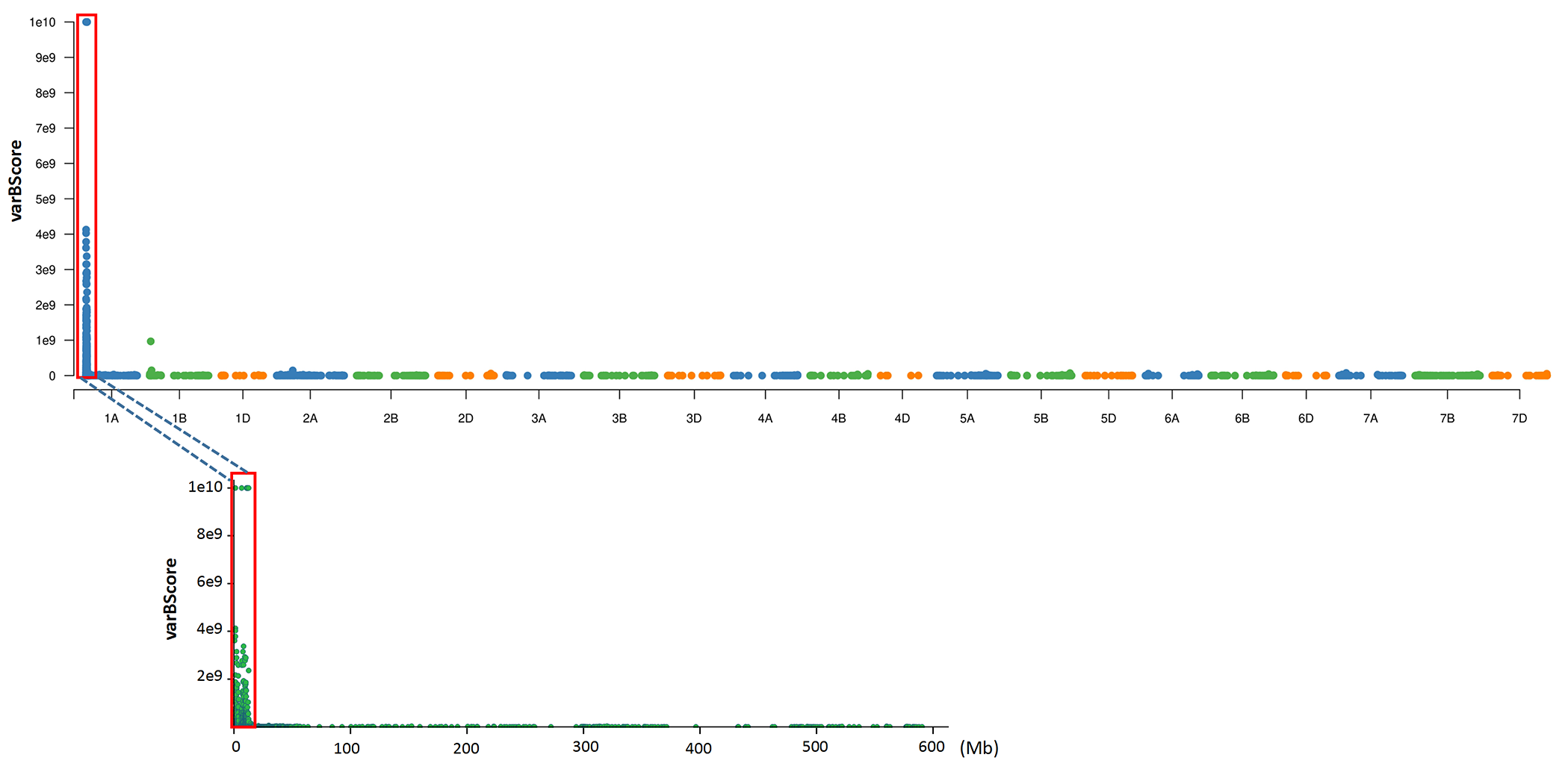

Supplement: SUPPLEMENTARY FIGURE S1 — Result of varBScore analysis for BSE-Seq. The above and below figures were drawn based on the varBScore of the variable sites of the whole genome and chromosome 1A, respectively. [file Image_1.TIF]

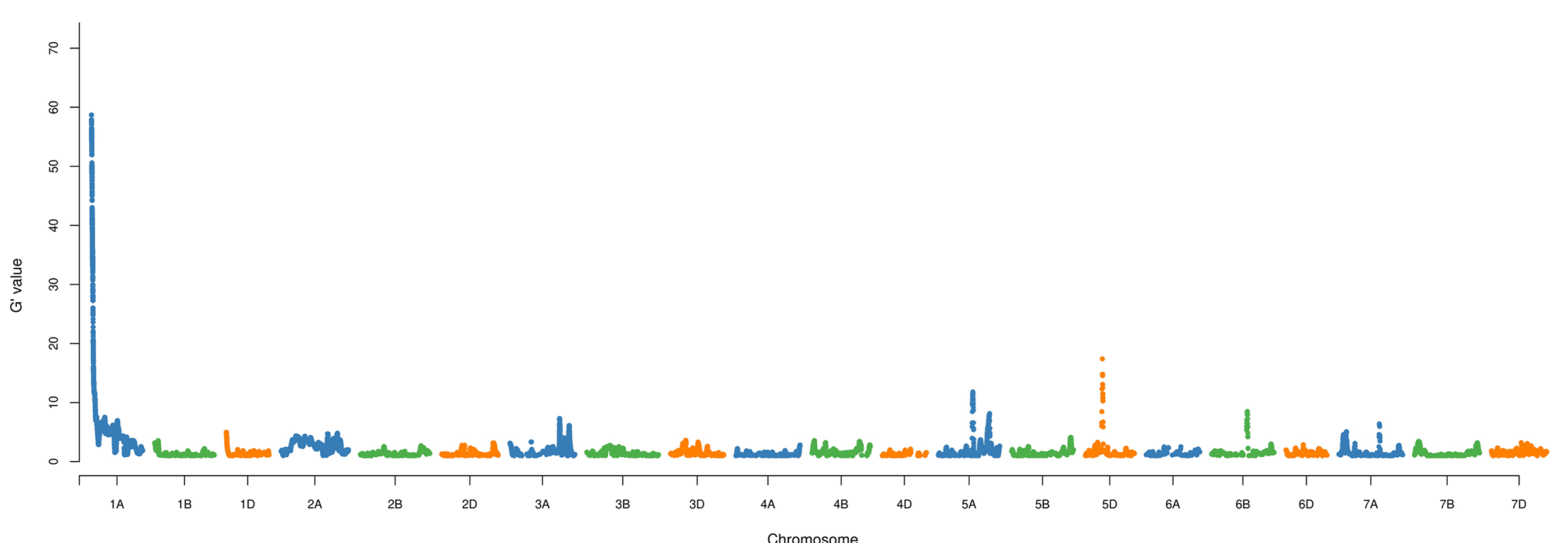

Supplement: SUPPLEMENTARY FIGURE S2 — Tricube-smoothed G’ value for BSE-Seq. [file Image_2.TIF]

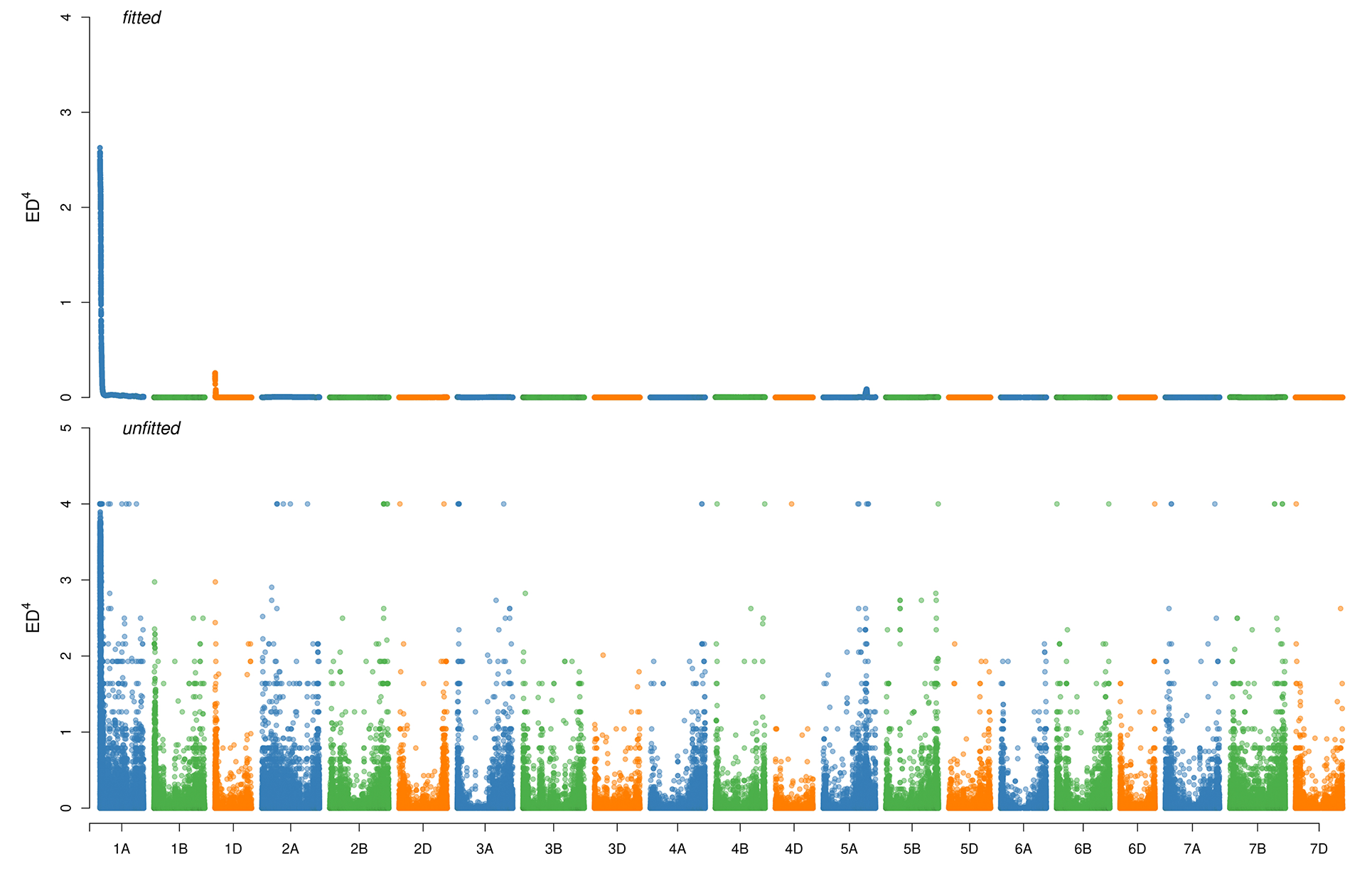

Supplement: SUPPLEMENTARY FIGURE S3 — Result of ED analysis for BSE-Seq. The above and below figures were drawn based on the fitted curve and original value, respectively. [file Image_3.TIF]

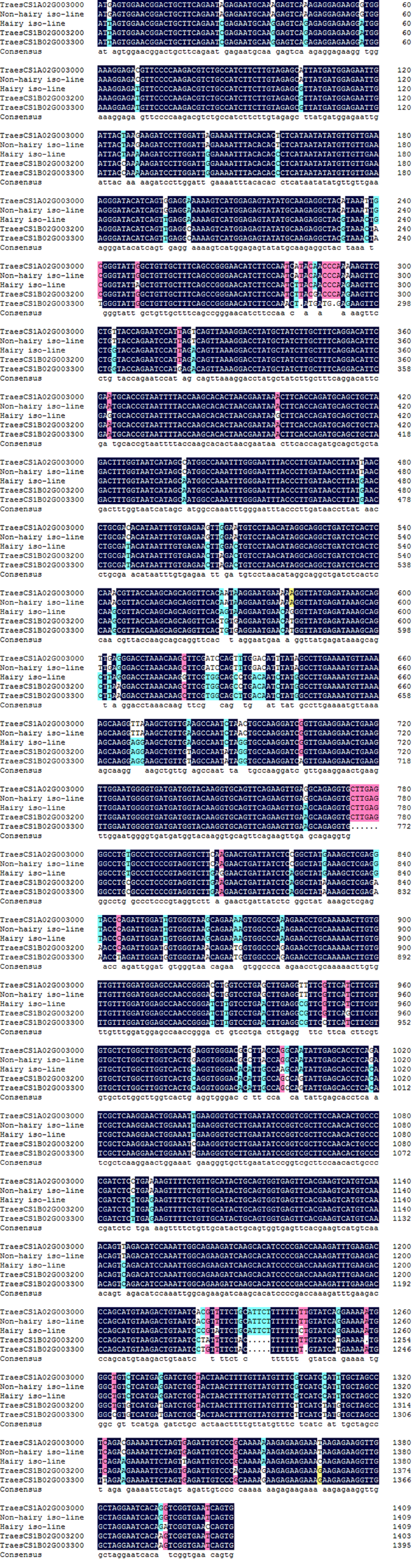

Supplement: SUPPLEMENTARY FIGURE S4 — Sequence comparison of amplified TraesCS1A02G003000 in NIL1. [file Image_4.TIF]
